# The influence of meteorological variables on the oviposition dynamics of *Aedes aegypti* (Diptera: Culicidae) in four environmentally distinct areas in northeast Brazil

**DOI:** 10.1590/0074-02760200046

**Published:** 2020-07-10

**Authors:** Isabella Cristina da Silva Santos, Cynthia Braga, Wayner Vieira de Souza, André Luiz Sá de Oliveira, Lêda Narcisa Regis

**Affiliations:** 1 Fundação Oswaldo Cruz-Fiocruz, Instituto Aggeu Magalhães, Programa de Pós-Graduação em Saúde Pública, Recife, PE, Brasil; 2 Fundação Oswaldo Cruz-Fiocruz, Instituto Aggeu Magalhães, Departamento de Parasitologia, Recife, PE, Brasil; 3 Fundação Oswaldo Cruz-Fiocruz, Instituto Aggeu Magalhães, Departamento de Saúde Coletiva, Recife, PE, Brasil; 4 Fundação Oswaldo Cruz-Fiocruz, Instituto Aggeu Magalhães, Núcleo de Estatística e Geoprocessamento, Recife, PE, Brasil; 5 Fundação Oswaldo Cruz-Fiocruz, Instituto Aggeu Magalhães, Departamento de Entomologia, Recife, PE, Brasil

**Keywords:** Aedes, meteorological concepts, time series studies

## Abstract

**BACKGROUND:**

Fluctuations in climate have been associated with variations in mosquito abundance.

**OBJECTIVES:**

To analyse the influence of precipitation, temperature, solar radiation, wind speed and humidity on the oviposition dynamics of *Aedes aegypti* in three distinct environmental areas (Brasília Teimosa, Morro da Conceição/Alto José do Pinho and Dois Irmãos/Pintos) of the city of Recife and the Fernando de Noronha Archipelago northeastern Brazil.

**METHODS:**

Time series study using a database of studies previously carried out in the areas. The eggs were collected using spatially distributed geo-referenced sentinel ovitraps (S-OVTs). Meteorological satellite data were obtained from the IRI climate data library. The association between meteorological variables and egg abundance was analysed using autoregressive models.

**FINDINGS:**

Precipitation was positively associated with egg abundance in three of the four study areas with a lag of one month. Higher humidity (β = 45.7; 95% CI: 26.3 - 65.0) and lower wind speed (β = −125.2; 95% CI: −198.8 - −51.6) were associated with the average number of eggs in the hill area.

**MAIN CONCLUSIONS:**

The effect of climate variables on oviposition varied according to local environmental conditions. Precipitation was a main predictor of egg abundance in the study settings.

*Aedes aegypti*, an arthropod of the Culicidae family, subgenus Stegomyia is the main vector responsible for the rapid spread and intensive transmission of arboviruses, such as dengue, Chikungunya and more recently Zika worldwide.^1^ Recent studies analysing the distribution of *Ae. aegypti* in time and space point to the risk of expansion of this species to other regions of the world as a result of climate change.^2^ In addition, greater human mobility, including sea and air cargo transport, has favored the global expansion of this mosquito, even in temperate climate zones.^3^

There is strong evidence of the effect of climate variations, primarily temperature and precipitation, on the abundance of *Ae. aegypti* and, consequently, on the transmission dynamics of arboviruses such as dengue.^4^ ^,^ ^5^ High temperatures have effect on the survival, development rate, mortality and spread of *Aedes* species while high humidity is associated with increased *Ae. aegypti* feeding activity survival and egg development.^4^ ^,^ ^6^ Precipitation accelerates population growth of *Aedes* through the formation of new breeding sites, but when remarkably high can wash out the containers and negatively affect the abundance of these vectors.^7^ Wind speed either favors mosquito dispersal or suppresses their flight activity interfering with feeding habits and oviposition.^8^ Solar radiation may interfere with the egg-laying behavior of females, although studies present discordant results regarding the preference of females for shade or breeding sites exposed to the sun.^4^ It has been demonstrated that fluctuations in climate and local environmental conditions, such as level of urbanisation, are associated with variations in mosquito abundance and arbovirus incidence (in general, dengue) between settings.^9^

The Northeast region of Brazil has been affected by severe and repeated outbreaks of dengue and, more recently, Zika and Chikungunya, all diseases transmitted by *Ae. aegypti* ^10^ given the local effect of climate variations and physiographic characteristics on the population dynamics of *Ae. aegypti* (with consequent increased risk of transmission), it is important to investigate the population dynamics in different settings, with a view to establishing early warning systems and improving vector control measures in this region. In the present study, we examined the association between a set of meteorological variables (precipitation, temperature, humidity, wind velocity and solar radiation) and the oviposition dynamics of *Ae. aegypti* in four environmentally distinct urban settings in the State of Pernambuco, an area hyperendemic for arboviruses in Brazil.

## MATERIALS AND METHODS

**Fig. 1:**
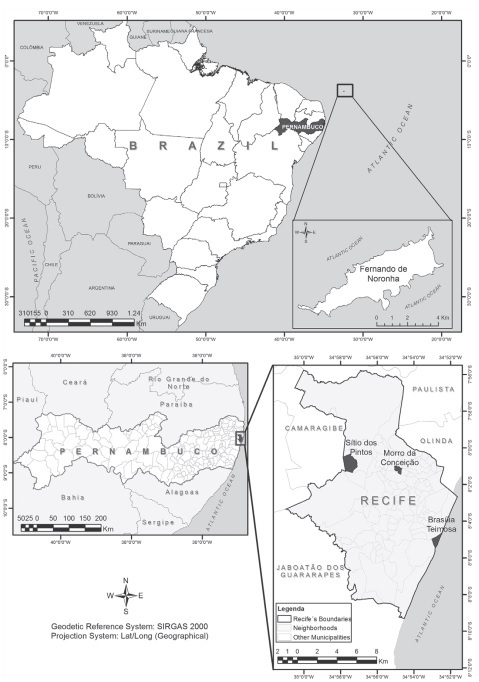
location of the study areas.

*Study design and setting* - A time series study was carried out using the databases created by one study conducted in the city of Recife (between April 2004 and May 2007),^11^ and one carried out in the Fernando de Noronha Archipelago (between January 2011 and May 2013),^12^ both in the State of Pernambuco in the Northeast region of Brazil (Fig. 1). The projects were reviewed and approved by the Research Ethics Committee of the Aggeu Magalhães Research Center ― Fiocruz Brazil (process: No. 14/04; CAAE No. 0095.0.095.000.10). Both studies were conducted with the aim of obtaining entomological parameters for an entomological surveillance system based on continuous collection of *Ae. aegypti* eggs by way of a sentinel ovitrap network (SMCP-Aedes - *Aedes aegypti* Monitoring and Population Control System).

The city of Recife (08º03’14”S; 34º52’52”W), capital of the State of Pernambuco, has a territorial area of 218.4 km^2^ and a population of about 1.6 million inhabitants, corresponding to a population density of 7,039.6 inhabitants/ km^2^.^13^ The regional climate is hot and humid with average annual temperatures around 25ºC, with higher averages in the months of January and February (≈ 27ºC) and lower ones in June and July (≈ 24ºC). Annual mean precipitation is 2,305 mm, with rainy seasons between April and July, and November and December are the driest months. Annual relative air humidity varies between 70% and 90% and the velocity of winds during the year ranges from 230 m/s to 340 m/s, with an annual average of 290 m/s. The archipelago of Fernando de Noronha (3º50’25 “S; 32º24’38” W) is composed of 21 islands in the Atlantic Ocean with a total area of 26 km^2^. Like the city of Recife, the climate is tropical humid with clearly defined dry and rainy seasons and a marked irregularity in interannual precipitation. The average temperature, of around 25ºC, varies little throughout the year and is slightly higher between November and April (≈ 30ºC) and slightly lower from May to October (≈ 28ºC). The average annual precipitation is 1,275 mm, with rainy seasons between February and July and drier seasons between October and November. The average speed of the winds is 660 m/s with the highest intensity between July and August. The relative humidity is around 80%. In Recife, the entomological data were collected in three urban sites with distinct physiographic characteristics: Brasília Teimosa, Morro da Conceição/Alto José do Pinho and Dois Irmãos/Sítio dos Pintos. In Fernando de Noronha, the data collection was carried out in 15 villages with size ranging from 14 to 172 households. Table I shows the main characteristics of the selected settings.

**TABLE I:** Main characteristics of the study settings, Pernambuco State, Brazil

Characteristics	Study areas			
	Brasília Teimosa	Morro da Conceição/ Alto José do Pinho	Dois Irmãos/ Sítio dos Pintos	Archipelago of Fernando de Noronha
Urban landscape	Coastal area	Hill	Suburban area located near a remnant of Atlantic forest.	Marine biome
Territorial area (km^2^)	0.61	0.38	1.80	17.017
Total Population	18,320	10,169	7,275	2,630
Population density (inhabitants/km^2^)	30,257.8	26,490.5	4,048.9	154.5
Households connected to the water supply network (%)	82.7	98.8	74.7	96.2
Households connected to the sewage network (%)	63.6	14.2	20.4	73.0
Households with proper garbage disposal (%)	99.6	99.0	97.3	100.0

Source: IBGE (2010).

*Data collection* - A detailed description of *Ae. aegypti* egg collection has already been provided.^12^ A total of 368 geo-referenced S-OVTs (100 in Morro da Conceição/Alto José do Pinho, 80 in Brasília Teimosa, 85 in Dois Irmãos/Sítio dos Pintos and 103 in Fernando de Noronha) were installed at fixed points in the external parts of the residences one meter above ground level, in the shade and protected from rain. In Fernando de Noronha the S-OVTs were spatially distributed according to the number of households in each village: five in villages with 14 to 30 households seven to eight in villages with 56 to 89 households and 10 in villages with more than 100 households. Therefore, the number of S-OVTs varied from 63 to 129 S-OVT per km² in the different settings. The collection and exchange of the S-OVTs palletes for egg counting was carried out weekly in around 25 S-OVT per area, on a rotating basis. In Recife eggs, were counted using a stereoscopic microscope with the aid of a manual cell counter^12^ whereas in Fernando de Noronha, a semi-automatic computerised system was employed. This computerised system is based on an optical platform that generates a digital image of the palletes.^14^ The monthly average of eggs in each study area was obtained by dividing the total number of eggs collected by the number of OVTs inspected in their respective month. The Table II shows the frequency distribution of the average number of eggs per month.

**TABLE II:** Frequency distribution of the number of eggs collected monthly by ovitraps in the study areas, Pernambuco State, Brazil

Study areas	Mean	Median	Standard deviation	Minimum	Maximum
Brasilia Teimosa	1445.6	1371.9	659.7	509.2	2947.0
Morro da Conceição/Alto José do Pinho	931.0	881.8	423.5	223.1	1903.2
Dois Irmãos/Sítio dos Pintos	487.1	443.2	215.9	159.3	1023.2
Archipelago of Fernando de Noronha	133.4	125.2	66.7	36.5	297.2

*Meteorological data source* - Monthly averages on precipitation (mm), temperature (ºC) relative air humidity (%), wind velocity (m/s) and solar radiation (W/m²) refer to the entire territorial area of Recife and Fernando de Noronha and were obtained from the International Research Institute for Climate and Society (IRI) climate satellite data library. Data on precipitation were specific to each of the three sites in Recife, while the data for Fernando de Noronha refer to the entire territorial area of the archipelago.^15^

*Data analysis* - Data analysis was performed using the GRETL (Gnu Regression, Econometrics and Time-series Library) and R version 3.2.4 programs. First the frequency distribution of the meteorological variables and the number of *Ae. aegypti* eggs in each study setting were described. Time trend graphs were generated to evaluate the stationarity of the time series (no trend). The temporal dependence structure of the stationary series was analysed using autocorrelation functions, which provide moving averages (q) and partial autocorrelation (p) and indicate autoregression. The effect of the meteorological variables on the average number of eggs was analysed using an autoregressive model of order 1 (AR1). The association of each meteorological variable with the average number of eggs was estimate through first order autoregressive term - AR(1) model and using different lags (lag 0, 1, 2 e 3).

$Y_{t}=\;c+\;{\varphi Y}_{\left(t-1\right)}+\;X\beta$

The selection of the meteorological variables in the final model was defined based on the use of the stepwise backward technique and the Akaike Information Criterion (AIC).^16^

## RESULTS

**Fig. 2:**
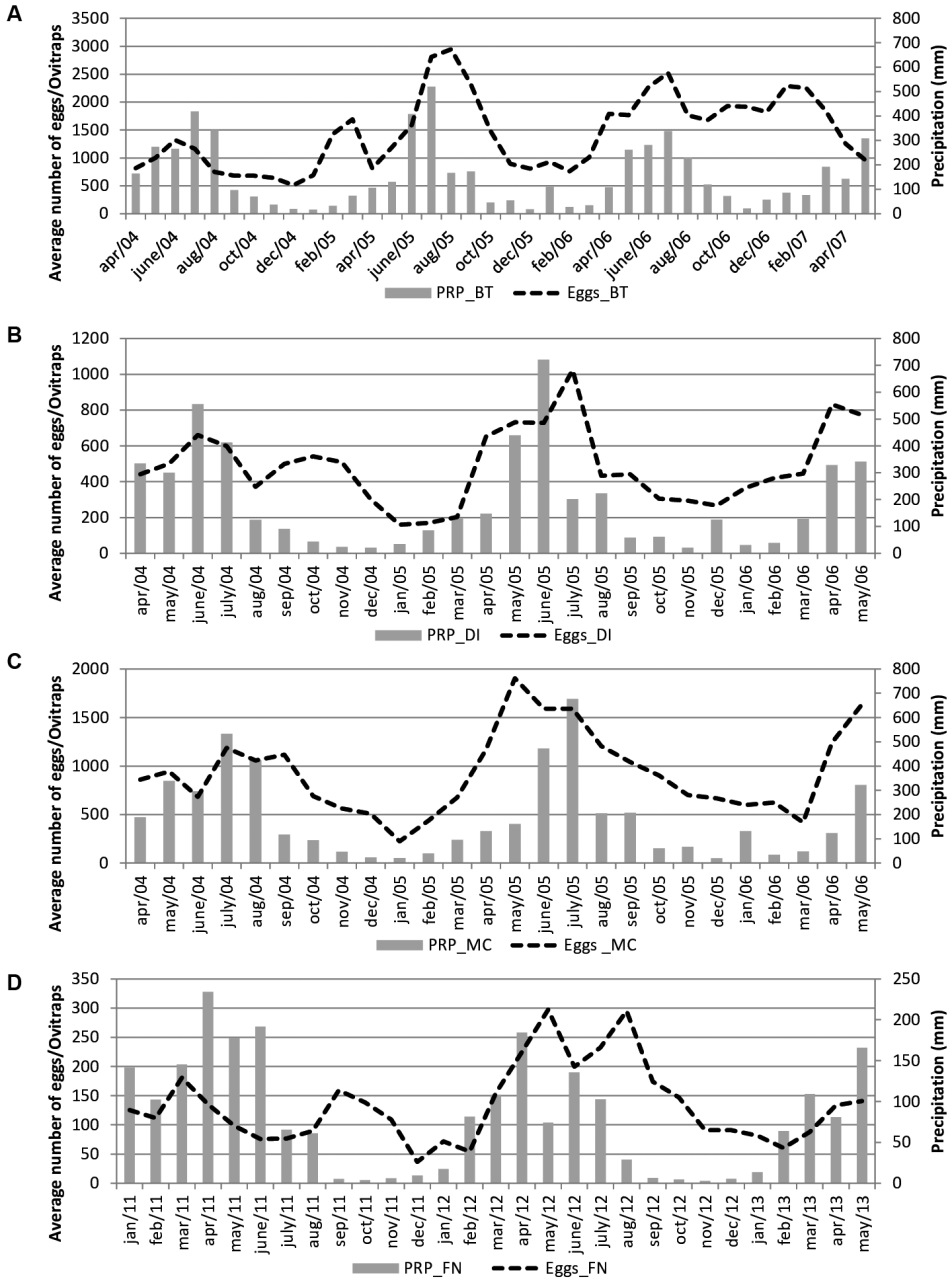
monthly variation in the number of eggs and precipitation in the study areas. (A) Brasília Teimosa (BT); B: Dois Irmãos/Sítio dos Pintos (DI); C: Morro da Conceição/Alto José do Pinho (MC); D: Fernando de Noronha Archipelago (FN).

*Descriptive analysis of meteorological and entomological data* - The Fig. 2 and Table II show the monthly variation in precipitation (mm) and average number of eggs collected in all study areas. In Brasília Teimosa, Morro da Conceição/Alto José do Pinho, Dois Irmãos/Sítio dos Pintos and Fernando de Noronha, the mean monthly precipitation was 155.3 mm, 190.4 mm, 189 mm and 80.3 mm, respectively, during the study period. In all areas of Recife, peak precipitation occurred in July 2005, while the driest period was between November and February. In Fernando de Noronha, peak precipitation occurred in April 2011 (234 mm) and there were lower precipitation averages between September and December.

**Fig. 3:**
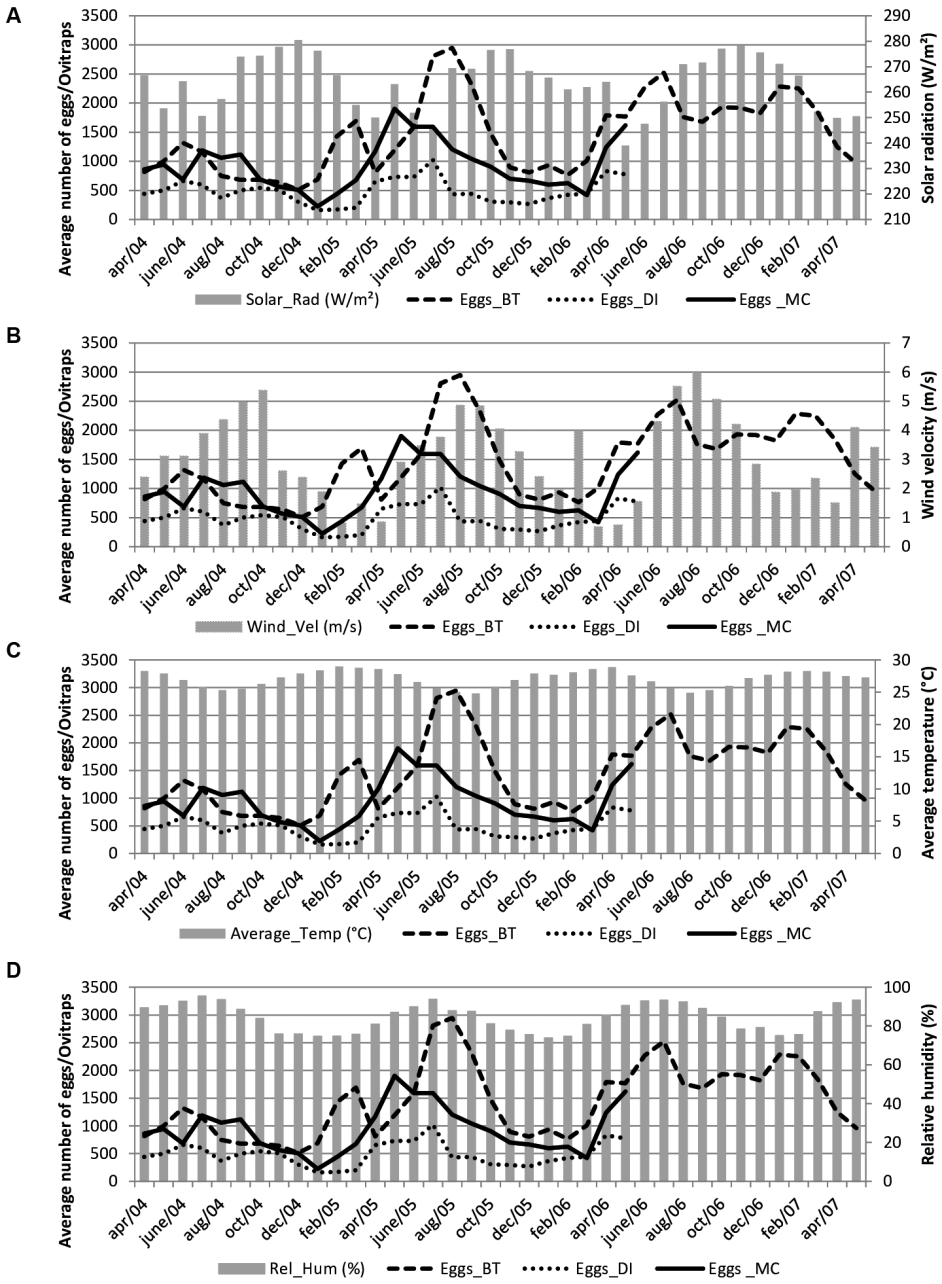
monthly variation in number of eggs and meteorological variables in Recife neighborhoods between April 2004 and May 2007. (A) solar radiation; (B) wind velocity; (C) average temperature; (D) relative humidity. Brasília Teimosa (BT); Dois Irmãos/Sítio dos Pintos (DI); Morro da Conceição/Alto José do Pinho (MC).

**Fig. 4:**
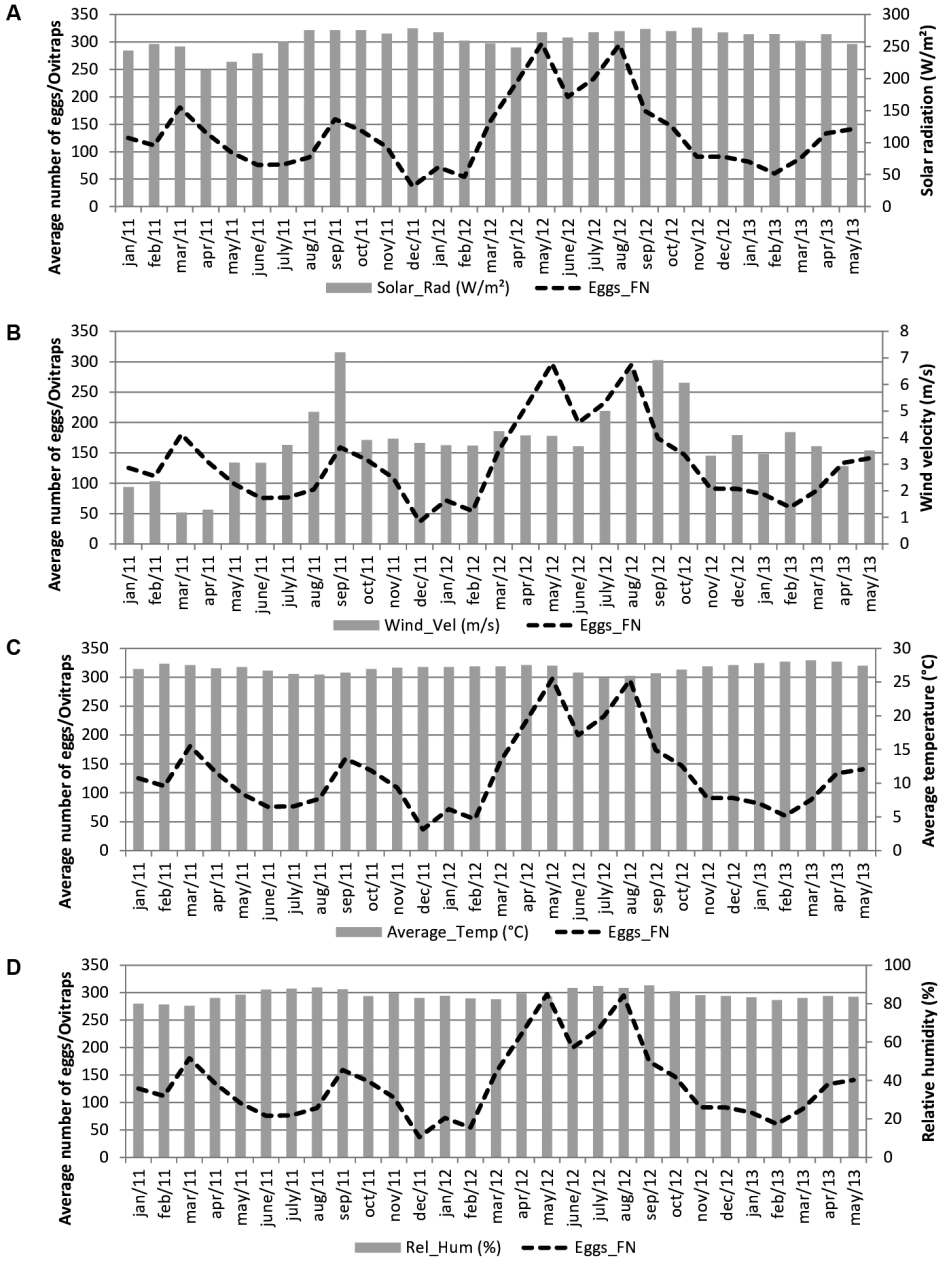
monthly variation in number of eggs and meteorological variables in the Fernando de Noronha Archipelago between January 2011 and May 2013. (A) solar radiation; (B) wind velocity; (C) average temperature; (D) relative humidity.

The monthly average temperatures, of around 27ºC (range 28ºC -26ºC), and the relative humidity, of around 80%, showed little variation throughout the year in either Recife or Fernando de Noronha. The monthly average solar radiation for all areas in Recife was 264 W/m², with the highest intensity in December 2004 (280.5 W/m²) and the lowest in May 2006 (239 W/m²) (Fig. 3A). In Fernando de Noronha, monthly solar radiation was 262 W/m² with a peak in November 2012 (279 W/m²) and lowest intensity in April 2011 (214 W/m²) (Fig. 4A).

The monthly average wind velocity in Recife was 3.1m/s with the lowest velocity in March 2006 (0.6 m/s) and highest in August 2006 (5.9 m/s) (Fig. 3B). In Fernando de Noronha, the monthly average wind velocity was 3.9 m/s with a minimum of 1.1 m/sin March 2011 and a maximum of 7.1 m/s in September 2011 (Fig. 4B).

The entomological data were as follows. The average numbers of *Aedes* eggs collected by month in the four study settings in Recife were 1,445.0 ± 659.7 in Brasília Teimosa, 931.0 ± 423.5 in Morro da Conceição and 487.1 ± 215.9 in Dois Irmãos/Sítio dos Pintos, with higher averages between April and August. In Fernando de Noronha, the monthly average number of eggs was 133.4 ± 66.7 with higher levels between March and August (Figs 2, 3, 4).

**Fig. 5:**
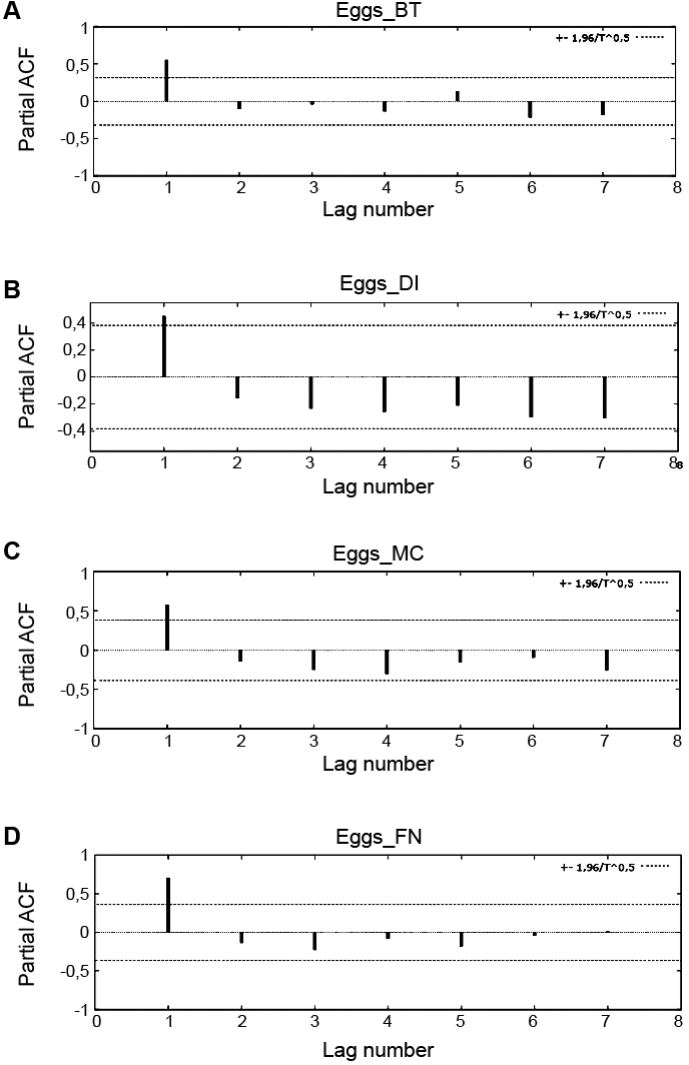
partial autocorrelation functions (PACFs) for the study areas. (A) Brasília Teimosa; (B) Dois Irmãos/Sítio dos Pintos; (C) Morro da Conceição; (D) Archipelago of Fernando de Noronha.

*Autocorrelation* - The partial autocorrelation functions for the average number of eggs per S-OVT showed that the egg averages in a given month had a strong correlation with the abundance of eggs for the previous month (Fig. 5). The autocorrelograms show p = 1 and q = 0 corresponding to a first-order autoregressive model (p) of the time series for the number of eggs. The stationarity of the time series for eggs (outcome) was tested and not rejected there by eliminating the differentiation process.

*Autoregressive models AR (1) of the association of meteorological variables with average number of Ae. aegypti eggs* - The Table III shows the adjusted estimates of the autoregressive models per study area. The time lags used in the model were those found in the analysis of the partial autocorrelation function of each meteorological variable with the egg averages. The time lags used in the model were those found in the analysis of the partial autocorrelation function of each meteorological variable with the egg averages. Precipitation remained positively associated with the abundance of eggs in Brasília Teimosa [β = 1.36; 95% confidence interval (CI): 0.04 - 2.68], Dois Irmãos (β = 0.69; 95% CI: 0.31 - 1.08) and in the Fernando de Noronha Archipelago (β = 0.38; 95% CI: 0.02 - 0.73). The humidity was positively associated with the average number of eggs only in Morro da Conceição/Alto José do Pinho (β = 45.68, 95% CI: 26.3 - 65.0), while wind speed remained inversely associated with the eggs only in Morro da Conceição/Alto José do Pinho (β = -125.2; 95% CI: -198.8 - -51.6).

**TABLE III:** Final autoregressive models AR (1) of the association between meteorological variables and number of *Aedes aegypti* eggs in the study setting of Recife and the Fernando de Noronha Archipelago. Pernambuco, Brazil

Final model	Study areas											
	Brasília Teimosa			Morro da Conceição/Alto José do Pinho			Dois Irmãos/Sítio dos Pintos			Fernando de Noronha Archipelago		
	Coefficient (95%CI)	p	AIC	Coefficient (95%CI)	p	AIC	Coefficient (95%CI)	p	AIC	Coefficient (95%CI)	p	AIC
AR1	0.74 (0.5 - 0.9)	0.000	557.7	0.66 (0.36 - 0.95)	0.000	346.9	0.42 (0.08 - 0.79)	0.021	328.6	0.68 (0.42 - 0.93)	0.000	301.4
Precipitation (*Lag* 1)	1.36 (0.04 - 2.68)	0.042		-	-		0.69 (0.31 - 1.08)	0.000		0.38 (0.02 - 0.73)	0.033	
Solar radiation (*Lag*0)	-	-		-	-		-	-		-	-	
Wind speed (*Lag* 1)	-	-		−125.2 (−198.8 - 51.6)	0.000		-	-		-	-	
Temperature (*Lag* 0)	-	-		-	-		-	-		-	-	
Humidity (*Lag* 0)	-	-		45.68 (26.3 - 65.0)	0.000		-	-		-	-	
Constant	1,190.6 (678.2 - 1,702.9)	0.000		−2.531 (−4,195.6 - 866.5)	0.002		361.3 (236 - 486.7)	0.000		101.5 (44.5 - 158.4)	0.000	

AIC: akaike information criterion; CI: confidence interval.

## DISCUSSION

The analysis showed that precipitation was a predictor of *Ae. aegypti* eggs abundance in all study areas, except for Morro da Conceição/Alto José do Pinho, a densely populated and higher altitude neighborhood in the city of Recife. Lower wind speed and higher humidity were only associated with egg means per S-OVT in Morro da Conceição/Alto José do Pinho. Temperature and solar radiation were not associated with variations in the average number of eggs in any of the study areas.

The average number of eggs per S-OVT in the areas of Recife was markedly higher than the average number of eggs observed in Fernando de Noronha, suggesting a lower population density of *Ae. aegypti* in the latter area. This result confirms data from previous studies that showed a much higher abundance of eggs in cities of Pernambuco where the same monitoring method was used, when compared to the averages obtained for Fernando de Noronha.^11^ ^,^ ^12^ In addition to climatic factors, some peculiarities of Fernando de Noronha, such as its geographical characteristics, low population density and urban conformation, may limit the dispersion and reproduction of *Aedes*. It is also worth mentioning that *Ae. aegypti* was introduced in Fernando de Noronha later than in Recife. Instead, the city of Recife, with high population density and poor urban infrastructure and sanitation, has much more favorable conditions for the development of breeding and reproductive activity of this mosquito. On the other hand, the city of Recife has a high population density and large areas with poor urban and sanitation infrastructure that favor the development of breeding sites and proliferation of this mosquito.

Embryonic development period, hatching time of larvae, and development of immature forms, as well as the (extrinsic and intrinsic) incubation period of *Ae. aegypti*, are all parameters that influence the definition of the time lag in time series models.^17^ Therefore, the observed variation in time lag between meteorological variables possibly explained by the differences in their effect on the distinct phases of the *Aedes* biological cycle.

According to data from several studies carried out in different regions of the world,^18^ ^,^ ^19^ ^,^ ^20^ the analysis showed that precipitation was positively associated with the abundance of eggs in all areas, with the exception of Morro da Conceição/Alto José do Pinho. These results suggest that the occurrence of precipitation is one important factor that contributes to the increase of egg density in the study settings. The effect of precipitation on population growth of *Ae. aegypti* has mostly been related to local environmental characteristics, especially the availability and diversity of containers able to retain rainwater in the local environment.^18^ ^,^ ^19^ ^,^ ^20^ Another possible explanation for the increase in the vector population would be the massive hatching of live eggs in the environment after the occurrence of precipitation.^11^ ^,^ ^12^ It is reasonable to assume that both mechanisms may have contributed to the increase in egg production after the rains in the studied areas. In the area of Dois Irmãos/Sitio dos Pintos, with less population density and greater vegetation cover, it is possible that the second mechanism (hatching of live eggs) played a more important role.

Air temperature is one of the most important climatic variables that influence physiology, behavior, ecology and, by extension, insect survival.^21^ Surprisingly, unlike the results obtained in other studies conducted in other region of Brazil,^17^ no association was found of the temperature with egg production in the studied areas. In these settings, as in the entire Northeast region of Brazil, the temperature averages are between 20**º**C and 28**º**C throughout the year, levels of temperature considered ideal for the development and reproduction of *Aedes*.^21^ Therefore, we suppose that this extremely low variability of this climatic data at ideal levels (26º - 28^o^C) for *Aedes* reproduction favors the maintenance of high population densities of this insect in the region throughout the year. Perhaps this fact explains the lack of seasonality of dengue transmission in our study setting, as observed by Cortez et al.,^22^ in a time series study comparing epidemiological data from Recife in Goiânia, in the Central-Western region of Brazil.

Similar phenomenon may have occurred for air humidity, which, like temperature, has a recognised effect in increasing the longevity of adult forms of *Aedes* species and facilitating blood-feeding, dispersion and egg-laying,^23^ ^,^ ^24^ ^,^ ^25^ ^,^ ^26^ but was associated with the abundance of eggs in only one of the studied areas. In Recife, the monthly average relative humidity is around 80% throughout the year reaching slightly higher levels between May and August (above 80%), which coincides with the rainy season in the region. These levels of air humidity (as temperature), which are considered ideal for *Aedes* reproduction ,in combination with the slight variation throughout the year, may explain the lack of association between this meteorological variable and egg production in most of the areas studied.

In our study, we found a negative association between wind speed and egg abundance in Morro da Conceição/Alto José do Pinho. These results are different from previous studies conducted in the city of Colombo, Sri Lanka,^27^ and the State of Florida,^28^ where a positive association was observed between wind speed and egg production. Turbulence and wind speed are meteorological factors that can positively or negatively affect the development and reproduction of *Aedes*. It is assumed that higher wind speeds can favor the passive migration of mosquitoes and favor their flight over long distances from their breeding sites.^29^ The negative effect of wind speed in a single study setting may be associated with local particularities of the environment that would interfere with the effect of this variable on *Aedes* oviposition activity.

We did not find any association between solar radiation and *Aedes* oviposition in any study setting and this result is maybe explained by the low variation of this variable in the regions. However, data on the effects of solar radiation on egg production are scarce and its effect on *Aedes* oviposition activity may be attributed to the behavior of the mosquito, which prefers to lay eggs in the shade.^30^

As a limitation of the present study it is worth noting that other factors possibly involved in egg production, such as pattern of urbanisation population density socioeconomic factors (per capita income) and geographical characteristics, such as altitude and vegetation were not included in the analysis. Likewise, the relatively short observation period (three years) and the time interval between egg collections (monthly) may have adversely affected the accuracy of the estimates.

The use of two different egg counting methods (manual cell counter and semi-automatic computerised system) could also lead to variations in the estimation of parameters if there were differences in precision between them. However, a validation study of the semi-automatic method in relation to the manual did not show differences in the egg count in the examined pallets^14^ suggesting that this problem may not have occurred.

However, it is worth highlighting the advantages of the study design which allowed information to be obtained from areas with different geographical and environmental characteristics within the same region. Also noteworthy is the fact that meteorological variables were obtained from satellite data for each particular neighborhood there by enabling much more accurate analysis.

In summary the results suggest that the effect of precipitation on *Aedes* oviposition dynamics in the study areas. Moreover the climate characteristics and the narrow range of variation in meteorological variables in the region studied particularly for temperature and humidity make it ideal for the reproduction of the *Aedes* mosquito enabling continuous reproduction of the mosquito throughout the year and, consequently permanent transmission of arboviruses. We conclude that these meteorological data in statistical models to predict increases in the *Ae. aegypti* population may not be applicable in this region.
